# The Genomic Landscape of Lobular Breast Cancer

**DOI:** 10.3390/cancers13081950

**Published:** 2021-04-18

**Authors:** Amy E. McCart Reed, Samuel Foong, Jamie R. Kutasovic, Katia Nones, Nicola Waddell, Sunil R. Lakhani, Peter T. Simpson

**Affiliations:** 1 Centre for Clinical Research, The University of Queensland, Herston, Brisbane, QLD 4029, Australia; Samuel.Foong@health.qld.gov.au (S.F.); j.kutasovic@uq.edu.au (J.R.K.); s.lakhani@uq.edu.au (S.R.L.); 2 Pathology Queensland, Royal Brisbane and Women’s Hospital, Herston, Brisbane, QLD 4029, Australia; 3 QIMR Berghofer Medical Research Institute, Herston, Brisbane, QLD 4006, Australia; Katia.Nones@qimrberghofer.edu.au (K.N.); Nic.Waddell@qimrberghofer.edu.au (N.W.)

**Keywords:** invasive lobular carcinoma, breast cancer, lobular, genomics, sequencing, precision oncology

## Abstract

**Simple Summary:**

We present a meta-analysis of invasive lobular carcinoma (ILC) sequencing data to provide a unified resource for ILC research. A large amount of data has been generated, but remains siloed due to the application of different sequencing approaches and limitations around cohort size and clinical annotation. To enact the goals of precision oncology in the field of lobular breast cancer, a substantive reference point is required, which we present herein. Furthermore, with combined datasets, we were able to define the prognostic significance of relevant clinico-pathology features.

**Abstract:**

Invasive lobular carcinoma (ILC) is the second most common breast cancer histologic subtype, accounting for approximately 15% of all breast cancers. It is only recently that its unique biology has been assessed in high resolution. Here, we present a meta-analysis of ILC sequencing datasets, to provide a long-awaited ILC-specific resource, and to confirm the prognostic value and strength of association between a number of clinico-pathology features and genomics in this special tumour type. We consider panel (*n* = 684), whole exome (*n* = 215) and whole genome sequencing data (*n* = 48), and review histology of The Cancer Genome Atlas cases to assign grades and determine whether the ILC is of classic type or a variant, such as pleomorphic, prior to performing statistical analyses. We demonstrate evidence of considerable genomic heterogeneity underlying a broadly homogeneous tumour type (typically grade 2, estrogen receptor (ER)-positive); with genomes exhibiting few somatic mutations or structural alterations, genomes with a hypermutator phenotype, and tumours with highly rearranged genomes. We show that while *CDH1* (E-cadherin) and *PIK3CA* mutations do not significantly impact survival, overall survival is significantly poorer for patients with a higher tumour mutation burden; this is also true for grade 3 tumours, and those carrying a somatic *TP53* mutation (and these cases were more likely to be ER-negative). Taken together, we have compiled a meta-dataset of ILC with molecular profiling, and our analyses show that the genomic landscape significantly impacts the tumour’s variable natural history and overall survival of ILC patients.

## 1. Introduction

Invasive lobular carcinoma (ILC) is the second most commonly diagnosed breast cancer histologic subtype, and accounts for equivalent numbers of patients as either ovarian cancer or triple-negative breast cancer [1]. Defined by a lack of cellular cohesion through the loss of E-cadherin, ILC is typified by a single-file infiltrating growth pattern [1]. However, annotation of ILC morphological variants is increasingly being made, and more data are emerging on the solid, alveolar, and pleomorphic subtypes, among others. Although most commonly estrogen receptor (ER)-positive and HER2-negative, ILC can also be either ER-negative, HER2-positive or triple negative (TN; negative for ER, progesterone receptor and HER2). Emerging data suggest, in fact, that the TN-ILC are a unique, aggressive group of tumours that are more likely to present in patients of older age and to be of the luminal androgen receptor subtype [2]. In spite of clear biological differences (e.g., sites of metastases), ILC is currently managed in the clinic in the same fashion as grade and stage-matched invasive breast cancer of no special type (IBC-NST). While prognosis over the short term is good, it is increasingly clear that ILC patients do worse than IBC-NST (grade-dependent) over time [3,4], and are more likely to metastasize to unusual sites, such as gynaecological and gastrointestinal organs, than IBC-NST [5,6,7].

High resolution ILC genomic research has emerged over the last five years (reviewed in [8]), and the value of ILC sub-studies in clinical trials is undoubted. In order to facilitate detailed analysis of ILC, in the context of precision oncology, we present a meta-analysis of available ILC genomic datasets to develop a unified resource to inform further essential ILC research.

## 2. Results and Discussion

### 2.1. A Unified ILC Cohort?

Bringing together the work of several different studies, we standardised where possible (see Methods) and present the clinical data and summarised exome (TCGA [9], *n* = 217) and panel-based (METABRIC [10,11], *n* = 192; EURO [12], *n* = 413; RATHER, [13], *n* = 79) mutation data from 901 ILC cases across multiple cohorts in Table S1. Given the challenges of conducting comprehensive cohort-based studies such as these, each cohort has its own limitations. Notably, the TCGA dataset lacks pathology-confirmed ER/PR/HER2 status and histological grade information, and the EURO dataset lacks PAM50 annotation. By accessing H&E sections of the ILC cases within the TCGA pan-cancer set through the Genomic Data Commons Data Portal, we were able to assign a grade to 145 cases and to assign histological variant classifications where possible (*n* = 197/217). The classification of morphological variants was very limited for METABRIC, while for the EURO set, detailed classification was made into solid, alveolar, and trabecular types, with the pleomorphic type grouped into a ‘mixed-non classic’ subtype along with ILC with other cytological variants (e.g., signet ring, apocrine, histiocytoid) [12]. There was overlap between the RATHER and METABRIC cohorts; therefore, we merged the data without duplicating cases.

There were notable cohort specific distributions of clinicopathology features (Table 1). For instance, 169/192 METABRIC cases were grade-annotated with 98/169 (57.9%) grade 2, while 106/145 (73%) assessable TCGA cases were grade 2. There was therefore a significant enrichment for grade 3 cases in the METABRIC set (*χ*^2^; *p* = 0.0009), and this skewing of the ILC population within METABRIC is likely a consequence of the eligibility criteria for the study (e.g., tumour size and tumour cellularity for molecular investigation).

### 2.2. Mutation Profiles of ILC

**Figure 1:**
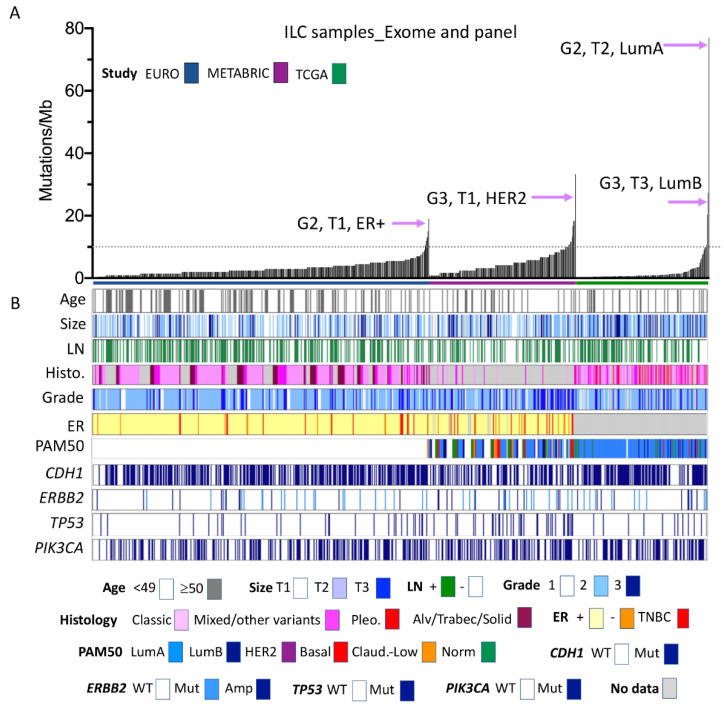
Clinical, morphological, and molecular characteristics of ILC cohorts analysed by gene panel and exome sequencing. (**A**) Individual ILC from three separate cohorts are ordered along the *x*-axis by increasing mutation burden (*y*-axis, calculated according to the number of mutations detected per Mb of the genome sequenced). Tumour features (grade, size, and tumour phenotype) of those ILC with the highest mutation burden in each cohort are highlighted. (**B**) Clinical and pathological features of the tumours sequenced, and mutations detected in common driver genes in ILC. Size: <2 cm = T1; 2–5 cm = T2; >5 cm = T3. Alv, alveolar; Amp, amplified; Clas., classic; ER, oestrogen receptor status; LN, lymph node; Mix., mixed and other variants; Mut, mutated; TN-ILC, triple negative ILC; Sol., solid; Trabec, trabecular; WT, wild type; G2, grade 2; G3, grade 3; LumA/LumB, Luminal A/B phenotype; Claud., claudin-low.

As described by early cohort studies ([12,13,14], reviewed in [8,15]), the most commonly altered driver mutations in ILC are in *CDH1*, *TP53*, *PIK3CA*, *FOXA1*, *PTEN*, *TBX3*, *FGFR2*, *ERBB2*, and *ERBB3*. *AKT1* and *CTNNA1* are increasingly important in ILC [16,17]; however, relatively low mutation frequencies were detected when profiled. These data are presented in Table 1 and Figure 1, and together with detailed clinicopathologic information in Table S1. Here, we investigated the distribution of these alterations across the different cohorts, and then we used the combined cohort to investigate the associations between various pathological and genomic features (i.e., grade, ER status, gene mutations and tumour mutation burden).

As shown in Table 1, the distribution of mutations in *CDH1, TP53, ERBB2, BRCA1/2* and *TBX3* differs across the four cohorts. A combination of sequencing technologies, different variant-calling pipelines, cellularity requirement for sample inputs, as well as regional population differences likely account for these variations. Importantly, *CDH1* alterations were variably reported across the cohorts (37–64%), being highest in the EURO set (*p* < 0.001). Significant enrichments for alterations in *TP53* (*p* < 0.001) and *ERBB2* (*p* = 0.0084) were noted for the METABRIC cohort, as a likely consequence of there being more grade 3 tumours in this dataset. Overall, *ERBB2* alterations were present in approximately 10% of the ILC cases, supporting recent work from Memorial Sloane Kettering [18,19], which also pointed to an enrichment for *ERBB2* alterations in the metastatic setting of ILC, as did [20,21]. There was no significant difference in *PIK3CA* mutation frequency between cohorts.

Given the cohort size and the high number of grade 3 and ER-negative tumours, we observed significant associations (Chi-squared/Fisher’s exact tests; Table S2; Table 2) between both increasing grade and ER-negative status and the enrichment of *TP53* (*p* < 0.00000001) and *ERBB2* mutations (*p* < 0.02). Interestingly, there was a significant association between the absence of *CDH1* mutations and ER negativity (*p* = 0.0204).

Figure 1 shows ILC cases studied by panel and exome sequencing, annotated for various clinicopathological parameters. The cases are ordered according to the tumour mutation burden. To facilitate this comparison, we calculated a ‘somatic mutations per Mb’ score, using 50 Mb as the denominator for the TCGA exome study [9], 1.2 Mb for the METABRIC panel sequencing [11], and 2 Mb for the EURO panel [12]. The RATHER data were excluded here because this gene panel was not entirely ‘cancer driver’ focused (it was larger at 3.2 Mb and also included the kinome), and the overall mutation count for each case was, to the best of our knowledge, not reported. Each of the three presented cohorts demonstrate that most ILC have a low mutation burden, with >90% of cases not reaching 10 mutations/Mb, which is the FDA-approved cut-off for tumour mutation burden (Keynote-158; ClinicalTrials.gov Identifier: NCT02628067 https://clinicaltrials.gov/ct2/show/NCT02628067, accessed on 16 April 2021). The mutation burden was also associated with specific cohorts, with a significant increase in the number of higher mutation burden cases in both METABRIC and EURO cohorts compared to the TCGA cohort (*p* < 0.0000000001; Figure S1A). This is likely a function of the design and read depth differences between panels and exomes, although may also be a consequence of the grade skewing that is present in the METABRIC cases. Interestingly, in two of the three cohorts, the individual cases with the highest burden were grade 2 ILC (Figure 1).

In this meta-analysis, we see that grade and mutation burden are significantly positively correlated in ILC, with increasing grade being associated with increasing mutation burden (*p* = 0.0005; Table 2, Figure S1B). Higher mutation burden was also associated with ER-negative and triple negative states (Figure S1C; *p* = 0.0038), and with negative LN status (Figure S1D; *p* = 0.0038), older patient age at diagnosis (Figure S1E; *p* = 0.0001) and smaller tumour size (Figure S1F; *p* = 0.0241). A significant association between mutation burden and histologic subtype was also noted, with ILC of the solid type having the highest mutations/Mb (Figure S1G; *p* < 0.0001). *TP53*, *PIK3CA* and *ERBB2* mutations were significantly associated with mutation burden (Figure S1H, *p* = 0.0002; Figure S1I, *p* = 0.0012; Figure S1K, *p* < 0.0001), while there was no association with *CDH1* mutations (Figure S1J).

### 2.3. Prognostic Relevance of Genomic Alterations

**Figure 2:**
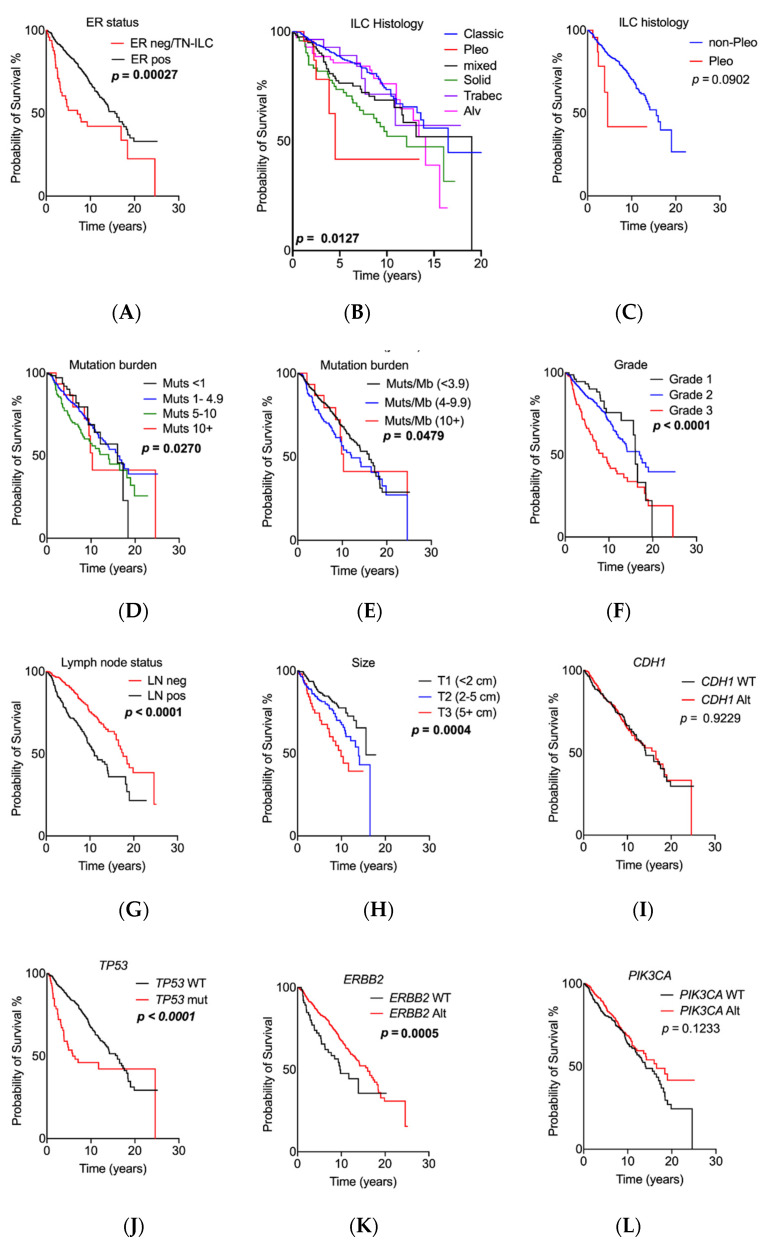
Association of genomic and pathology features with prognosis. Kaplan–Meier curves measuring survival associations of the following features: (**A**) overall survival; (**B**) ILC histology; (**C**) pleomorphic histotype; (**D**) grade; (**E**) lymph node status; (**F**) tumour size; (**G**) CDH1 gene status; (**H**) PIK3CA gene status; (**I**) TP53 gene status; (**J**) ERBB2 gene status; (**K**) ER status; and (**L**) tumour mutation burden expressed as mutations/Mb. The RATHER data were included for this analysis with the exception of (**L**). Alt, any genetic alteration; Alv, alveolar; AMP, amplification; LN, lymph node; MUT, mutation; neg, negative; pleo, pleomorphic; pos, positive; TN-ILC, triple-negative ILC; trabec, trabecular; WT, wild type. Significant *p*-values (Log-rank test) are noted in bold.

We performed Kaplan–Meier curve survival analysis to identify pathological and molecular features associated with prognosis across this large cohort (Figure 2). Here, we incorporated all cases with overall survival data rather than breast cancer-specific survival, because this was informative in more cases of the combined cohort. Figure 2A demonstrates that the cohort has a median survival time of 15.6 years, and that there is no significant difference between cohorts in terms of overall survival. Confirming previous findings [22], we show that the different histological subtypes of ILC confer differing prognoses, with pleomorphic ILC having the worst outcome, followed by solid types (Figure 2B,C). The caveat here is that pleomorphic ILC in the EURO set will be in the ‘mixed’ group, not in the pleomorphic ILC group. This merged cohort confirms that characteristic pathology indicators of grade (*p* < 0.0001), lymph node positivity (*p* < 0.0001) and tumour size (*p* = 0.0004) are each significantly prognostic in ILC ([4,23]; Figure 2).

Neither the *CDH1* nor *PIK3CA* gene mutation status had an impact on prognosis (Figure 2; *p* = 0.923 and *p* = 0.1233, respectively), while the presence of *TP53* mutations (*p* < 0.0001; Figure 2), and *ERBB2* alterations (mutations and amplifications) (*p* = 0.0222; Figure 2) were both associated with a significantly poorer outcome. This corroborates the recent meta-analysis of TCGA data reported by Kurozumi et al. [24], which showed a survival disadvantage in the presence of ERBB2 alterations, and contrasting with Deniziaut et al. [25], which reported no prognostic impact. We await the results of the SUMMIT trial (ClinicalTrials.gov Identifier: NCT01953926 https://clinicaltrials.gov/ct2/show/NCT01953926, accessed on 16 April 2021 [26]) and the mutHER trial (ClinicalTrials.gov Identifier: NCT01670877 https://clinicaltrials.gov/ct2/show/NCT01670877, accessed on 16 April 2021, among others) to determine the benefit of anti-HER2 therapy on a background of *ERBB2* mutations. The meta-analysis demonstrated that ER negativity also significantly impacts prognosis in ILC (*p* = 0.00000019, Figure 2), confirming the work of [27]. Given that only 21/757 ILC cases had a mutation burden of more than 10/Mb, we also considered the prognostic implications of a lower cut-off (<3.9 vs. 4–9.9 mutations/Mb), and we observed that an increasing mutation burden was significantly associated with poor outcome (*p* = 0.029; Figure 2).

### 2.4. What Can We Learn from the Whole Genome Data?

**Figure 3:**
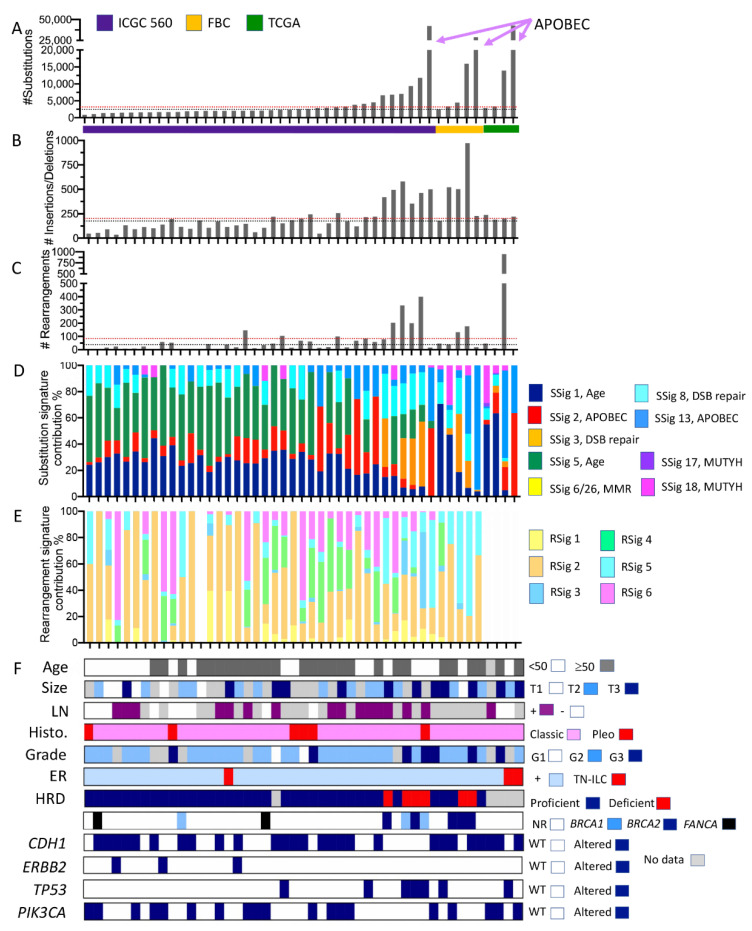
Somatic mutation characteristics of ILC derived from whole genome sequencing data. Individual tumours from three cohort studies are organised in each plot on the *x*-axis according to increasing numbers of somatic substitution mutations (*y*-axis in **A**). Plots (**B**,**C**) show the numbers of small insertions/deletions and large rearrangements, respectively. ICGC 560 overall cohort medians for numbers of substitutions, insertions/deletions and rearrangements are depicted by the red dotted lines in (**A**–**C**); similarly, the medians for the ILC in this pooled cohort are depicted by the black dotted line. Plots (**D**,**E**) show how the proportion of substitutions (from **A**) and rearrangements (from **C**) were assigned to substitution mutational signatures (SSig) or rearrangement signatures (RSig), respectively. Note that rearrangement signatures for ILC from TCGA cohort were not calculated. (**F**) Clinicopathological features and mutation of key cancer driver genes. DSB repair, double strand break repair; FBC, familial breast cancer cohort; G1, G2, G3, grade 1, 2 or 3, respectively; Germ. Mut, germline pathogenic mutation; HRD, Homologous recombination deficiency, as defined by HRDetect [28] (proficient = functional HR-based DNA repair; deficient = non-functional HR-based DNA repair); MMR, mismatch repair; LN, lymph node; NR, not recorded; Pleo, pleomorphic; RSig, rearrangement signature; SSig, substitution signature; T1, T2, T3, tumour size; WT, wild type. Colour coding is described in the associated legends for each plot.

As with pooling data from panel and exome sequencing, the interpretation of WGS data from disparate sources can be challenging. Here, we accessed cases and associated mutational data from (i) the International Cancer Genome Consortium (ICGC) project involving the sequencing of 560 whole genomes (*n* = 38 ILC) [28,29]; (ii) ILC sequenced as part of an in-house cohort of familial breast cancers (FBC; *n* = 5) [30], which involved mutation signatures calculated using the same COSMIC substitution signatures V2 and rearrangement signatures as the ICGC [28,29]; and (iii) ILC sequenced as part of TCGA (*n* = 4), for which data were re-mapped and analysed using the same in-house pipelines as used for the familial breast cancer cohort (Figure 3, Figure S2, Figure S3 and Table S3). Other studies have performed WGS on breast cancer cohorts that likely include ILC, but were not analysed and processed in a unified manner to the cases analysed herein, and so were not included. Here, we took the opportunity to investigate the patterns of somatic alterations present in ILC that were not necessarily detected by panel or exome sequencing, such as structural alterations (copy number changes and translocations) and mutational signatures. These data yield interesting insights into the aetiology of tumour development.

ILC genomes have long been regarded as ‘quiet’ with few copy number alterations, relative to other breast cancer genomes. Looking within the ICGC 560 cohort, there is a significant difference between ILC cases and non-ILC cases in the total numbers of structural rearrangements detected (*t*-test, *p* = 0.0022), but not insertions/deletions or substitutions (Figure S2). Indeed, as shown in Figure S2, non-ILC cases have a higher average and greater range in the numbers of rearrangements than ILC. Across the three ILC datasets, the median numbers of substitutions, insertions/deletions and rearrangements were 2513, 179 and 38, respectively (black lines on plots in Figure 3). These values are comparable to those of the non-ILC ICGC 560 set which has median values of 2330 substitutions, and 169 insertions/deletions, but which has a higher number of rearrangements (112; *t*-test *p* = 0.0022). Tumours with low levels of genome complexity were characterised by substitution mutation signatures previously correlated with aging [31] and reported in many different cancer types (signatures 1 and 5; green and dark blue annotations in Figure 3D).

There is a subset of cases with large-scale genomic changes across all three mutation categories (substitutions, insertions/deletions, and rearrangements), and it is important to note that this was observed across the three contributing cohorts. Those cases with high frequencies of insertions and deletions were significantly more likely to be grade 3 (Fisher’s exact test; *p* = 0.0478), however relationships between grade and substitution, and grade and rearrangements did not reach significance. No significant relationships were documented between genome status and ILC variant histology, although the numbers are small.

The tumours with the highest numbers of mutations in each cohort exhibited >25,000 substitutions, and these variants were categorised predominantly as C > T substitutions attributed to hyperactivity of the AID/APOBEC family of cytidine deaminases (substitution signatures 2 and 13). These tumours had very low structural genome complexity (negligible number of rearrangements) (Figure S3). Conversely, one tumour in the TCGA cohort exhibited an extraordinary number of structural rearrangements, with high numbers of inter-chromosomal translocations clustered between chromosomes 1, 11, 13 and 17, leading to the focal amplification of numerous genomic loci (Figure S3). Finally, six ILC, including 4/6 of the highly ranked (based on mutation burden) ILC in the ICGC 560 cohort and two in the FBC, exhibited high levels of substitutions, insertions/deletions, and rearrangements that were associated with prominent substitution signature 3 and rearrangement signatures 3 and 5 (Figure S3). These tumours harboured germline mutations in either *BRCA1* or *BRCA2*, and tumours were considered homologous recombination deficient according to HRDetect analysis [28,30].

### 2.5. Limitations

In an ideal world, a single large cohort of ILC (with treatment annotation) would be profiled using a single comprehensive platform to high depth, and the histopathology centrally reviewed. Although this has not yet been feasible, the ILC cohort papers published to date have advanced the field significantly. We noted some challenges with pooling the data together from different sources and different sequencing strategies.

The categorisation of ILC variants has evolved over time, and there are significant limitations and no absolute criteria for their classification. Indeed, in our experience, most ILC variants exhibit multiple variant types (e.g., solid and pleomorphic), as opposed to being a pure variant, akin to mixed metaplastic carcinomas, and therefore classification and associating clinical and/or genomic parameters to a single variant classification status may be limited. Nevertheless, the data compiled here and by others (e.g., [12]), clearly shows that certain variants are associated with defined genomic alterations or prognostic implications, and so we accept these limitations, and look towards a future, robust definition of ILC variant subtypes.

To account for the inherent limitations in cross-cohort analyses, we have restricted our analysis to a small set of driver genes, well covered by panels and exomes, in an effort to minimise the variability around sequence coverage. The high variability associated with different platforms and analysis tools for copy number alterations was a confounding factor, and as such we restricted our inclusion of copy number data only to the *ERRB2* gene. We have made an effort to carefully highlight findings while contextualising them within the background of this meta-analysis.

The long follow-up required for understanding the prognostic implications of the molecular findings and the difficulty in reporting historical treatment data means that the findings may not necessarily relate to the therapeutic strategies for current ILC patients.

## 3. Materials and Methods

### 3.1. Cohorts and Analysis

Samples were amassed for meta-analysis from a number of independent studies: TCGA (*n* = 215 exomes, *n* = 4 WGS) [9]; METABRIC (*n* = 192) [10,11]; EURO (*n* = 413) [12]; RATHER (*n* = 79) [13]; Familial Breast Cancer (*n* = 5) [30]; and ICGC 560 (*n* = 38) [29]. Due to the lack of mutation count information, RATHER data were omitted from Figure 1, however unique RATHER cases (not included in METABRIC dataset) were included in the association and survival analysis where informative data permitted. TCGA whole genome sequencing data were subjected to signature analysis according to the protocols detailed in [30], while for other cohorts, processed sequencing data were extracted and compiled. Data were audited, standardised where possible, and allocated into categorical bins. For example, tumour size was standardised to T1 (<2 cm), T2 (2–5 cm); T3 (>5 cm).

Data were analysed and graphs prepared using Prism v8.1. Kaplan–Meier curves were assessed for significance with a Gehan–Wilson test, with significance at *p* < 0.05, and considered overall survival. Associations were measured with a *χ*^2^ test, or Fisher’s exact test, with *p* < 0.05.

### 3.2. Pathology Review

Diagnostic haematoxylin and eosin-stained sections were accessed through the Genomic Data Commons Data Portal (https://portal.gdc.cancer.gov (accessed on 10 August 2020) for each TCGA ILC case, and reviewed by a pathologist (S.F.) for grade and histological subtypes, where possible. We recorded 3 cases wherein the diagnosis of the sample changed: 2 from ILC to IBC-NST, and 1 from mixed ductal lobular to ILC, on the basis of H&E morphology review. We retained the IBC-NST cases in Table S1 for transparency, but did not include them in analyses.

## 4. Conclusions

We present a meta-analysis of invasive lobular carcinoma genomic data in an effort to unify several important cohort studies. This meta-analysis highlights important variations or missing types of data across the analysed cohorts, which affect interpretations to some degree. Nevertheless, it is clear that while ILC exhibit recurrent mutations in *CDH1* and *PIK3CA* and have broadly quiet genomes, there persists a subset of ‘non-conforming’ ILC cases in which interesting genomic features are hidden beneath this curious morphological growth pattern.

*CDH1* mutations are pathognomonic for ILC and occur in the majority of tumours; loss of E-cadherin has been shown to unequivocally drive the ILC phenotype [17,32,33,34,35,36]. The frequency in which *CDH1* mutations are reported in lobular lesions in the literature is highly variable (42–82%, [12,13,14,15,37], and is highest in microdissected lobular in situ carcinoma lesions, 81–94% [38,39,40]), suggesting that mutation reporting is likely impacted by (i) the tumour cellularity of the individual specimens analysed, for what is described as a diffusely infiltrating tumour type; and (ii) the quality and sensitivity of sequencing technology used (panel, exome, whole genome). *PIK3CA* mutations are the second most common alteration in ILC, and these mutations may provide an important therapeutic target for patients. While the *PIK3CA* mutations do not impact prognosis in these historical cohorts not exposed to therapies targeting these mutations, this may change in the future with more widespread use of PI3K/MTOR inhibitors. Indeed, the recent study from Teo et al. [17] demonstrates that E-cadherin loss also activates the PI3K/Akt pathway, and this can occur independently of *PIK3CA* mutations. Together with data from [14] indicating the importance of *AKT* mutations (although infrequent), this shows the potential therapeutic importance of this pathway in ILC, as does the sensitivity of ILC models to the therapeutic targeting of AKT [17].

Beyond E-cadherin and PI3K pathways, the less frequent morphological and molecular features of ILC confer great interest, and these may account for the inter-tumour heterogeneity in the biological and clinical nature of this disease. These data confirm that ILC variant histology is prognostic [41], and that there is important value in undertaking a standardisation of the ILC variant classification. Once a clear guideline is established, it will be important for this to be reported diagnostically, and for the prognostic implications of specific variants and their potential relationships with treatment guidelines to be determined. Additional features that were associated with poor prognosis in the large case series studied by panel and exome sequencing included grade 3 ILC, an ER-negative phenotype, mutations in *TP53* or *ERBB2*, and a high tumour substitution mutation burden (>5 mutations/Mb). Indeed, these features were inter-related, as one might expect with what we understand about invasive breast cancer in general.

The whole genome sequencing meta-analysis further demonstrated that most ILC have low genomic complexity, with low numbers of substitutions, insertions/deletions, and rearrangements. These tumours would fit the classic 1q gain/16q loss ‘simple’ genomes previously associated with ILC [15]. However, in each dataset there are tumours with considerable genome complexity, which are associated with (i) a low number of structural rearrangements but a hypermutation genotype linked to APOBEC mutagenesis; or (ii) tumours with highly rearranged genomes affecting clustered sets of chromosomes, which would fit with a previous description of complex ‘firestorm’ rearrangements [42] and have been associated with complex high level amplifications (and indeed co-amplification) of 11q13 and 8p12 in ER-positive breast cancer and ILC [43,44,45]; or (iii) tumours exhibiting tumour genomes characteristic of homologous recombination (HR) DNA repair deficiency [28,30], which is associated with treatment opportunities involving platinum-based chemotherapy or PARP-inhibitors. It would be important to know whether these three mutational signature types are related to resistance to endocrine therapy or prognosis in ILC.

While we have restricted this analysis to early breast cancer, there are several large studies that provide detailed analysis of the genomics of advanced ILC. Indeed, applying WGS, [46] showed that metastatic breast cancers have a higher tumour mutation burden and that 52% of metastatic BC harbour actionable mutations. It is not clear what proportion of this cohort have lobular breast cancer. A recent analysis of endocrine-resistant breast cancers from Memorial Sloan Kettering included a large proportion of ILC assessed using various iterations of the MSK-Impact sequencing panel (132 metastatic ILC; 127 primary ILC [18,19]). The findings specific to metastatic ILC are consistent with those genomic features that were associated with poor prognosis in primary ILC: higher mutation burden and frequency of mutations affecting driver genes TP53 and ERBB2, as well as mutations in ESR1 [18]. These analyses also confirmed the prevalence of NF1 mutations, as an emerging mechanism of endocrine resistance [18,21], as well as FAT1, which confers resistance to CDK4/6 inhibitors when inactive [19,47]. It is clear that sequencing of advanced ILC provides significant opportunities for identifying treatment escape mechanisms, actionable alterations, and providing ILC patients with tailored treatments.

Taken together, these data and those of the collective large papers suggest that there are some very important morphological and molecular findings which could impact the variable clinical behaviour of ILC: grade 3, morphological variants, ER-negativity, alterations in *PIK3CA*, *TP53*, and *ERBB2*, and signatures associated with APOBEC, complex structural rearrangements, and HR deficiency. There needs to be a concerted effort to align these findings with clinical response to hormone therapy and outcomes in a large and unified collaborative, international study that combines detailed morphological classifications of ILC and its variants with a standardised genomic analysis to capture these diverse types of somatic mutations. Such an analysis would help to define an important set of morphological and molecular parameters to be reported diagnostically to inform future management.

In summary, we have provided a meta-analysis of a number of landmark genomic publications in an effort to centralise ILC data, to add important genomic insights into the biological and clinical nature of the disease, and to enable future research.

## Figures and Tables

**Table 1:** Relationships between invasive lobular carcinoma (ILC) pathology and molecular features across 4 cohorts.

Feature		Cohort (*n*; %)				*p*-Value (*χ*^2^)
		METABRIC *n* = 192	TCGA *n* = 215	EURO *n* = 413	RATHER *n* = 79	
Grade	1	17; 9%	20; 9%	47; 11%	4; 5%	*p* < 0.00000001
	2	106; 55%	146; 67%	301; 72%	69; 88%	
	3	58; 30%	31; 14%	63; 15%	1; 1%	
	N/A	11; 6%	20; 9%	2; 1%	5; 6%	
Histo-type	Classic	15; 8%	117; 54%	197; 48%	43; 54%	*p* < 0.0001
	Pleo.	0; 0%	29; 14%	0	0	
	Other/mixed	8; 4%	48; 22%	57; 14%	20; 25%	
	Solid	1; 1%	1; 1%	65; 15%	6; 8%	
	Trabec. *	0	0	28; 7%	0	
	Alveolar	1; 1%	0	66; 16%	4; 5%	
	N/A	167; 86%	20; 9%	0	6; 8%	
*CDH1* status	Mutated	88; 47%	110; 54%	265; 64%	29; 37%	*p* < 0.0001
	None reported	98; 53%	95; 46%	148; 36%	50; 63%	
*TP53* status	Mutated	15; 7%	35; 18%	30; 7%	3; 4%	*p* < 0.0001
	None reported	200; 93%	157; 82%	383; 93%	76; 96%	
*ERBB2* status	Mutated	10; 5%	35; 18%	30; 7%	3; 4%	*p* < 0.0001
	None reported	182; 95%	157; 82%	383; 93%	76; 96%	
*PIK3CA* status	Mutated	81; 38%	84; 44%	175; 42%	30; 38%	*p* = 0.453
	None reported	134; 62%	106; 56%	238; 58%	49; 62%	
*BRCA1* status	Mutated	2; 1%	2; 1%	2;1%	6; 8%	*p* = 0.00001
	None reported	184; 99%	208; 99%	411; 99%	73; 92%	
*BRCA2* status	Mutated	5; 3%	6; 3%	9; 2%	6; 8%	*p* = 0.0748
	None reported	183; 97%	201; 97%	404; 98%	73; 92%	
*ERBB3* status	Mutated	4; 2%	2; 1%	15; 4%	4; 5%	*p* = 0.13661
	None reported	186; 98%	209; 99%	398; 96%	75; 95%	
*FGFR2* status	Mutated	-	3; 1%	-	0; 0%	*p* = 0.2867
	None reported	-	208; 99%	-	79; 100%	
*FOXA1* status	Mutated	-	11; 5%	37; 9%	-	*p* = 0.1125
	None reported	-	204; 95%	376; 91%	-	
*PTEN* status	Mutated	5; 3%	12; 6%	18; 4%	2; 3%	*p* = 0.3317
	None reported	177; 97%	181; 94%	395; 96%	77; 97%	
*TBX3* status	Mutated	10; 5%	14; 7%	55; 13%	-	*p* = 0.0022
	None reported	172; 95%	201; 93%	358; 87%	-	
*AKT1* status	Mutated	4; 2%	5; 2%	17; 4%	2; 3%	*p* = 0.4606
	None reported	188; 98%	210; 98%	396; 96%	77; 97%	
*CTNNA1* status	Mutated	-	3; 2%	1; 1%	-	*p* = 0.3472
	None reported	-	189; 98%	214; 99%	-	

Note that not every case had data recorded for each feature; thus, the total number of cases changed for each feature. Regarding gene status, only mutations are included in this analysis, not copy number alterations. In the absence of data, we scored as none reported—this does not necessarily equate to wild type. * note that the WHO does not recognise trabecular as a histologic variant of ILC.

**Table 2:** Contingency table of relationship between ILC molecular features and grade across the combined cohort.

Combined Cohorts		TMB		*TP53* Status		*ERBB2* Status		*CDH1* Status		*PIK3CA* Status		PAM50					
		Low (1–9 mut/MB)	High (10+ mut/MB)	Altered	None Reported	Altered	None Reported	Altered	None Reported	Altered	None Reported	LumA	LumB	Basal	HER2	Claudin-Low	Normal-Like
**ER status**	**ER pos**	94	*5*	8	92	6	94	61	39	44	56	86	*7*	0	*1*	1	*5*
	**ER neg**	*77*	*23*	37	63	*25*	*75*	33	67	*37*	*63*	29	10	19	38	5	0
	**TN-ILC**	*85*	*15*	44	56	*19*	*81*	56	44	*37*	*63*	*9*	*0*	*18*	*27*	*37*	*9*
		*p* = 0.0460		*p* < 0.00000001		*p* = 0.0002		*p* = 0.0204		*p* = 0.6150		*p* < 0.00000001					
**Grade**	**Grade 1**	100	0	1	99	9	91	55	45	*52*	*48*	*88*	*0*	*0*	*1*	*4*	*7*
	**Grade 2**	97	3	7	93	7	93	64	36	45	55	*88*	*4*	*0*	*2*	*1*	*5*
	**Grade 3**	89	11	23	77	14	86	56	44	37	63	*63*	*21*	*4*	*7*	*2*	*3*
		*p* = 0.0073		*p* < 0.00000001		*p* = 0.0128		*p* = 0.1062		*p* = 0.0622		*p* < 0.00000001					

Table presented with percentages of cases as italic. Chi-squared *p*-values are in bold where significant. Detailed numbers of patients and proportions, as well the individual cohort analyses, are provided in Table S2.

## Data Availability

The data presented in this study are available in this article.

## Supplementary Materials

The following are available online at https://www.mdpi.com/article/10.3390/cancers13081950/s1. Figure S1: Whole exome and panel sequencing-derived mutation burden shows significant associations with clinical, pathology and genetic features. Figure S2: Comparison of the genomic catalogue between ILC cases, and non-ILC cases within the ICGC cohort. Figure S3: Circos plots depicting genomic architecture across representative samples from each of the three WGS cohorts. Table S1: Compiled dataset of whole exome and panel data. Table S2: Detailed breakdown of contingency analysis presented in Table 2. Table S3: Compiled dataset of whole genome sequencing data.
