# Correction: McCart Reed et al. The Genomic Landscape of Lobular Breast Cancer. *Cancers* 2021, *13*, 1950

**DOI:** 10.3390/cancers13164010

**Published:** 2021-08-09

**Authors:** Amy E. McCart Reed, Samuel Foong, Jamie R. Kutasovic, Katia Nones, Nicola Waddell, Sunil R. Lakhani, Peter T. Simpson

**Affiliations:** 1 Centre for Clinical Research, The University of Queensland, Herston, Brisbane, QLD 4029, Australia; Samuel.Foong@health.qld.gov.au (S.F.); j.kutasovic@uq.edu.au (J.R.K.); s.lakhani@uq.edu.au (S.R.L.); 2 Pathology Queensland, Royal Brisbane and Women’s Hospital, Herston, Brisbane, QLD 4029, Australia; 3 QIMR Berghofer Medical Research Institute, Herston, Brisbane, QLD 4006, Australia; Katia.Nones@qimrberghofer.edu.au (K.N.); Nic.Waddell@qimrberghofer.edu.au (N.W.)

The authors wish to make the following corrections to this paper [1]:

In the original published article, there were mistakes in Figure 1, Figure 2 and Figure 3. There were inconsistencies in labelling and a mismatch with the figure legends due to production issues during the proofreading of the paper. The corrected Figure 1, Figure 2 and Figure 3 appear below. 

The authors apologize for any inconvenience caused and state that the scientific conclusions are unaffected.

## Figures and Tables

**Figure 1:**
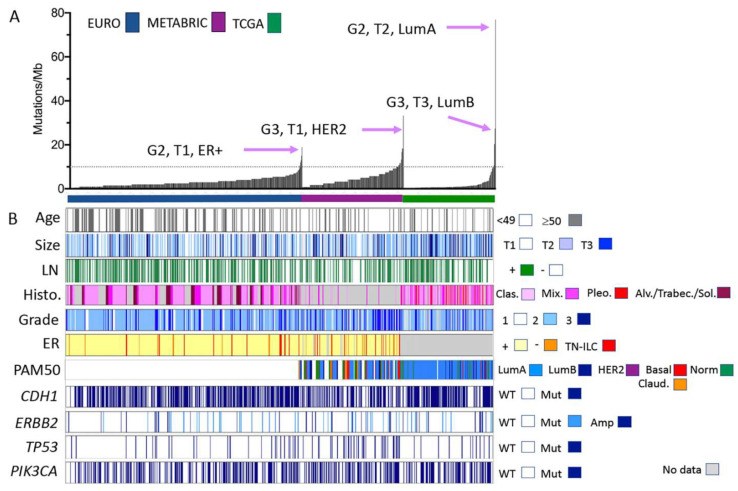
Clinical, morphological, and molecular characteristics of ILC cohorts analysed by gene panel and exome sequencing. (**A**) Individual ILC from three separate cohorts are ordered along the *x*-axis by increasing the mutation burden (*y*-axis, calculated according to the number of mutations detected per Mb of the genome sequenced). Tumour features (grade, size, and tumour phenotype) of those ILC with the highest mutation burden in each cohort are highlighted. (**B**) Clinical and pathological features of the tumours sequenced and mutations detected in common driver genes in ILC. Size: <2 cm = T1; 2–5 cm = T2; >5 cm = T3. Abbreviations: Alv = alveolar; Amp = amplified; Clas. = classic; ER = oestrogen receptor status; LN = lymph node; Mix. = mixed and other variants; Mut = mutated; TN-ILC = triple negative ILC; Sol. = solid; Trabec = trabecular; WT = wild type; G2 = grade 2; G3 = grade 3; LumA/LumB = Luminal A/B phenotype; and Claud. = claudin-low.

**Figure 2:**
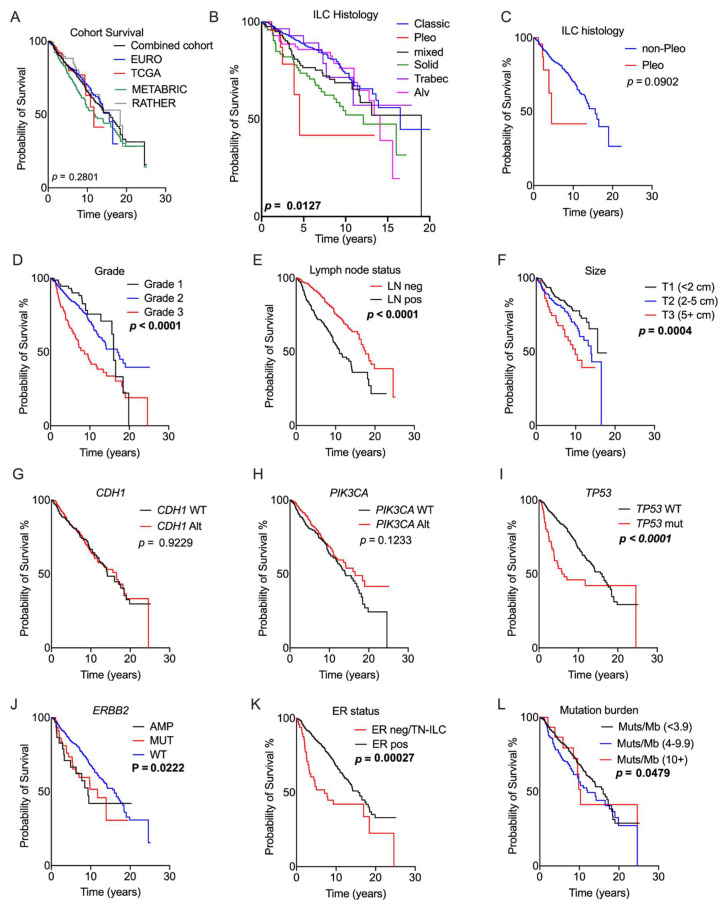
Association of genomic and pathology features with prognosis. Kaplan–Meier curves measuring survival associations of the following features: (**A**) overall survival; (**B**) ILC histology; (**C**) pleomorphic histotype; (**D**) grade; (**E**) lymph node status; (**F**) tumour size; (**G**) CDH1 gene status; (**H**) PIK3CA gene status; (**I**) TP53 gene status; (**J**) ERBB2 gene status; (**K**) ER status; and (**L**) tumour mutation burden expressed as mutations/Mb. The RATHER data were included for this analysis with the exception of (**L**). Abbreviations: Alt = any genetic alteration; Alv = alveolar; AMP = amplification; LN = lymph node; MUT = mutation; neg = negative; pleo = pleomorphic; pos = positive; TN-ILC = triple-negative ILC; trabec = trabecular; and WT = wild type. Significant *p*-values (log-rank test) are noted in bold.

**Figure 3:**
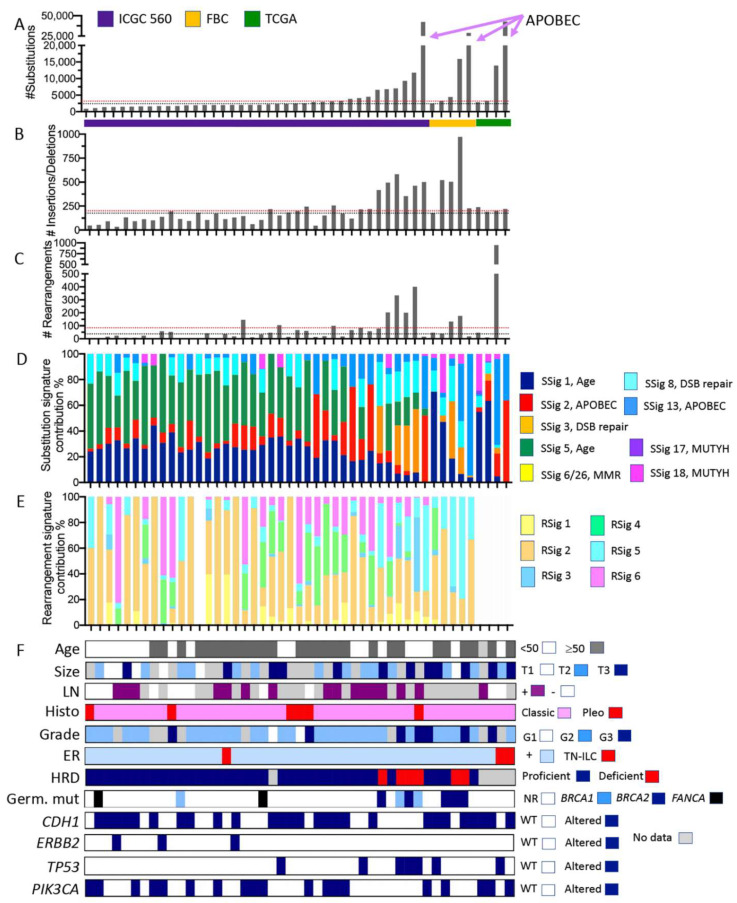
Somatic mutation characteristics of ILC derived from whole genome sequencing data. Individual tumours from three cohort studies are organised in each plot on the *x*-axis according to increasing numbers of somatic substitution mutations (*y*-axis in **A**). Plots (**B**,**C**) show the numbers of small insertions/deletions and large rearrangements, respectively. ICGC 560 overall cohort medians for the numbers of substitutions, insertions/deletions, and rearrangements are depicted by the red dotted lines in (**A**–**C**); similarly, the medians for the ILC in this pooled cohort are depicted by the black dotted line. Plots (**D**,**E**) show how the proportion of substitutions (from **A**) and rearrangements (from **C**) were assigned to substitution mutational signatures (SSig) or rearrangement signatures (RSig), respectively. Note that rearrangement signatures for ILC from the TCGA cohort were not calculated. (**F**) Clinicopathological features and mutation of key cancer driver genes. Abbreviations: DSB repair = double strand break repair; FBC = familial breast cancer cohort; G1, G2, G3 = grade 1, 2, or 3, respectively; Germ. Mut = germline pathogenic mutation; HRD = Homologous recombination deficiency, as defined by HRDetect [28] (proficient = functional HR-based DNA repair; deficient = non-functional HR-based DNA repair); MMR = mismatch repair; LN = lymph node; NR = not recorded; Pleo = pleomorphic; RSig = rearrangement signature; SSig = substitution signature; T1, T2, T3 = tumour size; and WT = wild type. Colour coding is described in the associated legends for each plot.
