# Supplementary material for: The Genomic Landscape of Lobular Breast Cancer
Source: Cancers (Basel). 2021 Apr 18;13(8):1950. doi: 10.3390/cancers13081950 (PMC8073944; doi:10.3390/cancers13081950)
Supplement: Supplementary file 1 [file cancers-13-01950-s001.zip › cancers-1163927-supplementary.pdf]

Supplementary Materials: The Genomic Landscape of Lobular Breast Cancer

Amy E. McCart Reed, Samuel Foong, Jamie R. Kutasovic, Katia Nones, Nicola Waddell, Sunil R. Lakhani and Peter T. Simpson

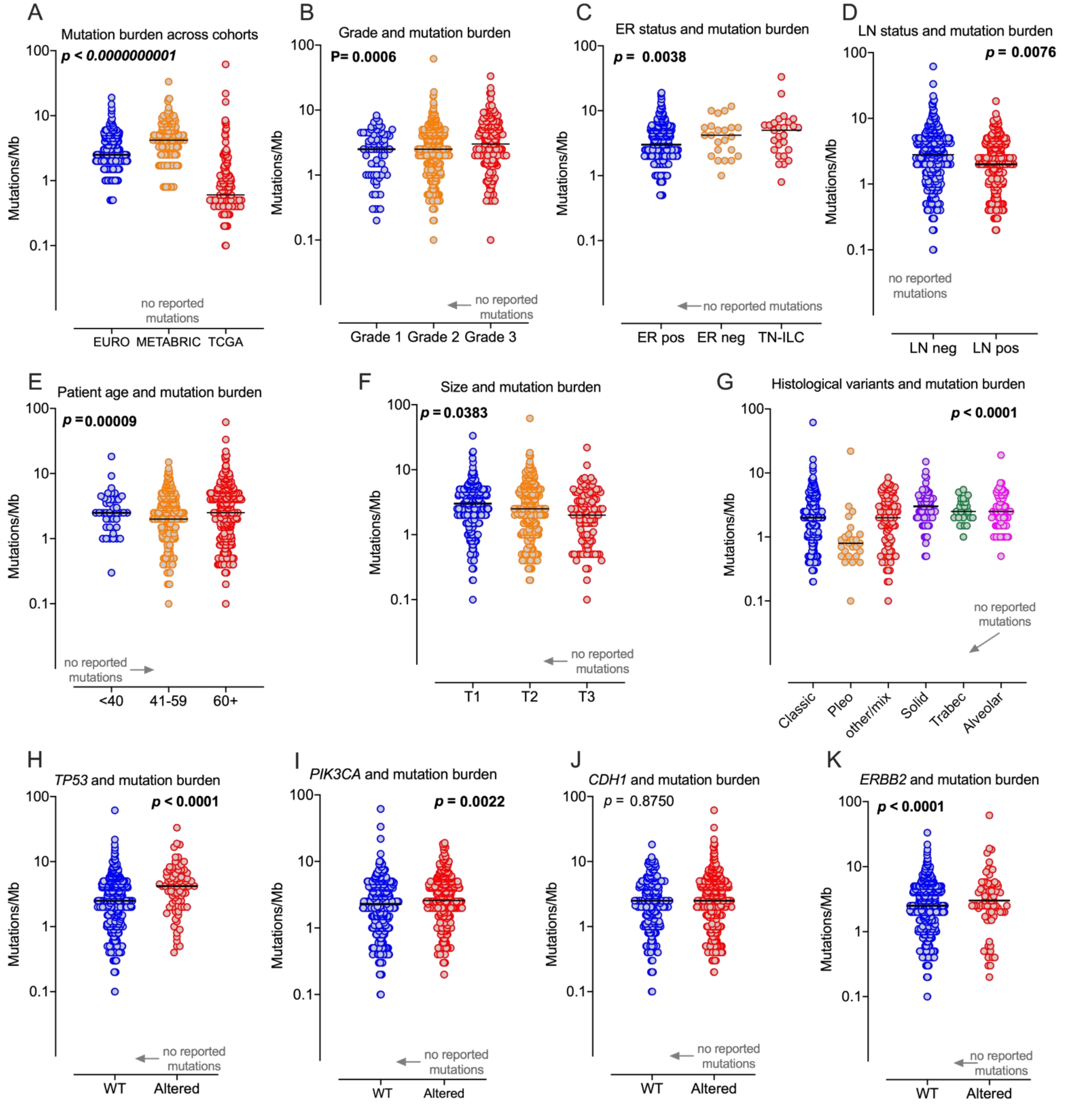

Figure S1. Whole exome and panel sequencing derived mutation burden shows significant associations with clinical, pathology and genetic features. (**A**) mutation burden across cohorts (note the exclusion of the RATHER cohort from this analysis due to the absence of TMB data; (**B**) grade; (**C**) oestrogen receptor status; (**D**) lymph node status; (**E**) patient age; (**F**) size, T1 < 2 cm, T2 = 2–5 cm, T3 > 5 cm; (**G**) histological variants; (H) *TP53* mutation status; (**I**) *PIK3CA* mutation status; (**J**) *CDH1* mutation status; (**K**) *ERBB2* mutation status. LN, lymph node; WT, wildtype/no mutation recorded.

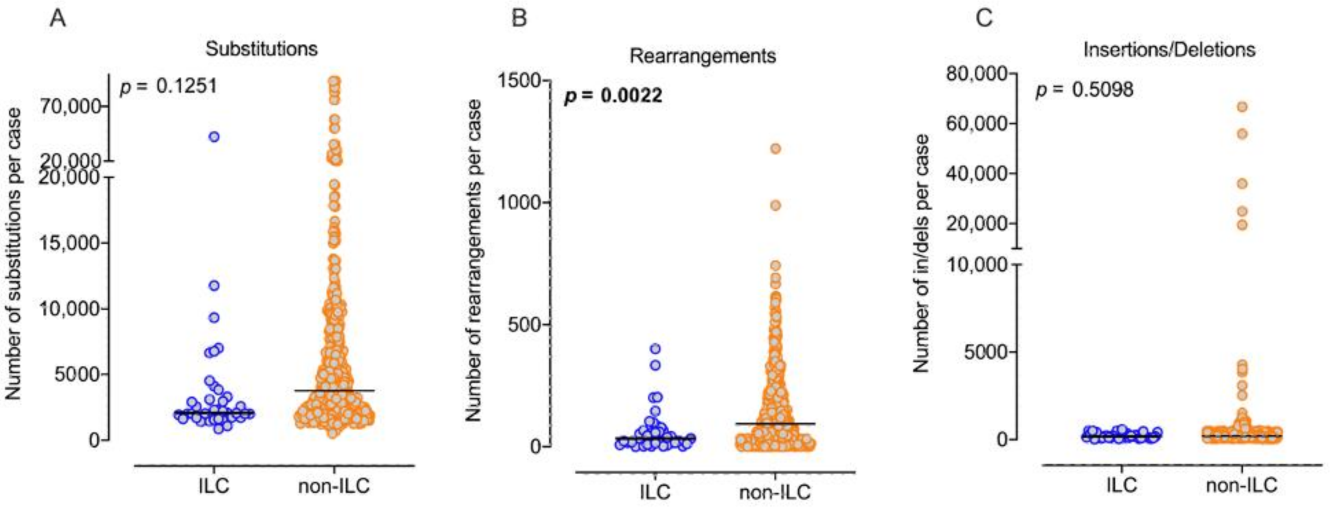

Figure S2. Comparison of the genomic catalogue between ILC cases, and non-ILC cases within the ICGC cohort. Shown from left to right are the (**A**) substitutions, (**B**) insertions/deletions and (**C**) rearrangements. Note the significant *p* values are in bold.

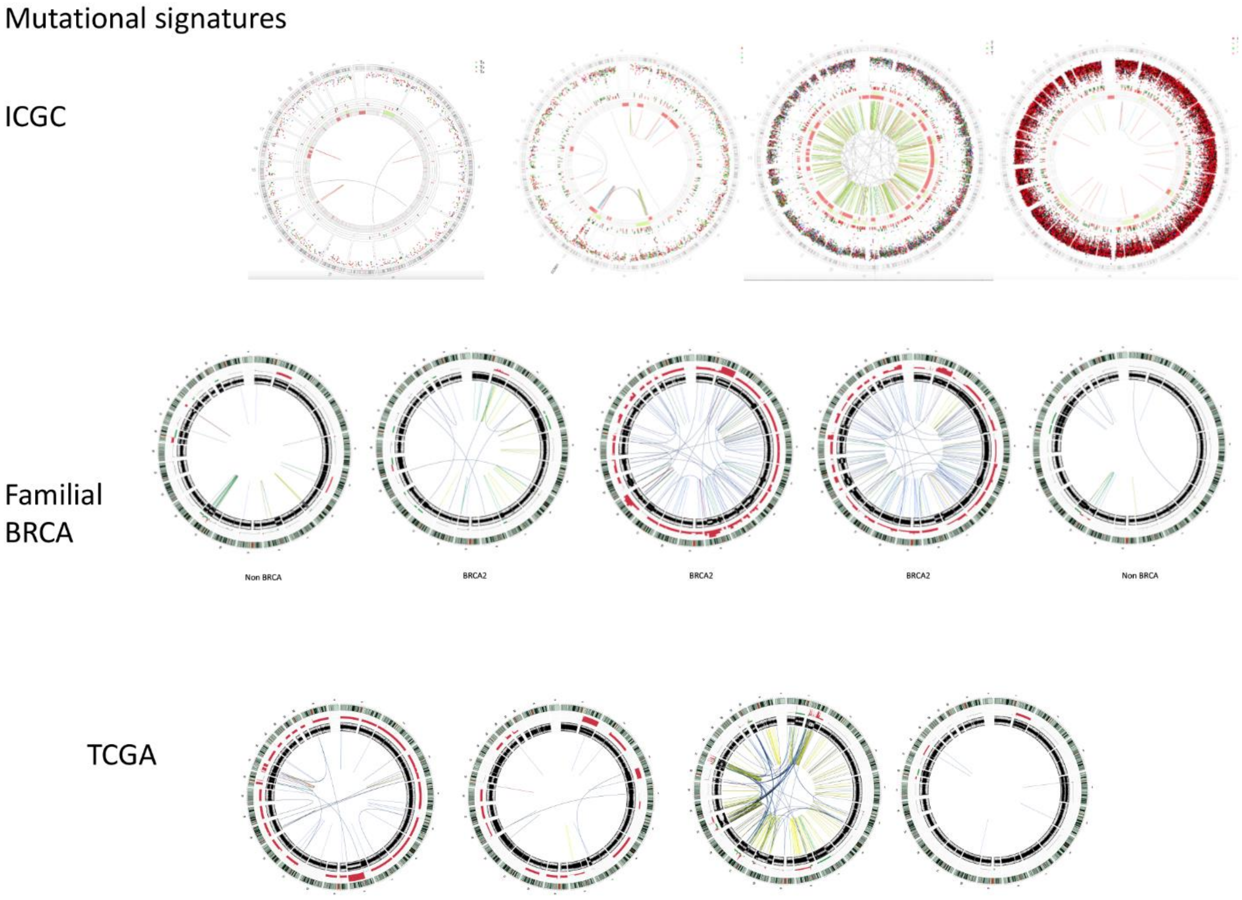

Figure S3. Circos plots depicting genomic architecture across representative samples from each of the three WGS cohorts. ICGC set: four ILC selected to demonstrate the heterogeneity of genome complexity in ILC. From left to right, cases show an increasing numbers of substitutions (colored dots around outside of plots); the second tumour has a complex rearrangement leading to co-amplification of 8p12 and 11q13; the third tumour has both a high mutation burden and a high rearrangement burden (as demonstrated by the complexity of rearrangements transiting through the centre of the plot); the fourth tumour has an extremely high substitution burden driven by APOBEC mutagenesis, but very low levels are structural alterations. These plots are screen grabs obtained from https://cancer.sanger.ac.uk/cosmic/sample/genomes. PD11767: ER+, PR+, HER2-, grade 2; PD6044: ER+, PR-, HER2-, pleomorphic ILC, grade 3; PD11748: triple negative,

pleomorphic ILC, grade 3; PD4977 triple negative ILC grade 3. Familial ILC set and TCGA set: showing all tumors considered in the analysis (Table S3).

Table S1. Compiled dataset of whole exome and panel data.

Table S2. Detailed breakdown of contingency analysis presented in Table 2.

Table S3. Compiled dataset of whole genome sequencing data.

Table S1–S3. are provided separately, attached as an Excel File.
